# Leptospirosis and Dengue Coinfection-Associated Multi-Organ Dysfunction Syndrome: A Case Report

**DOI:** 10.7759/cureus.52466

**Published:** 2024-01-17

**Authors:** Govind Shiddapur, Vutukuru Kalyan Kumar Reddy, Mohith Prakash Kondapalli, Saimounika Adapa, Sonali Agarwal

**Affiliations:** 1 General Medicine, Dr. D. Y. Patil College, Hospital and Research Centre, Dr. D. Y. Patil Vidyapeeth, Pune, IND

**Keywords:** coinfection, dengue hemorrhagic fever (dhf), dengue encephalitis (de), thrombocytopenia, dengue fever (df), leptospirosis

## Abstract

Dengue and leptospirosis are frequently discussed separately, with dengue causing rash and leptospirosis causing jaundice. Currently, there are more and more reports of coinfections. The comparable clinical symptoms of both infections make it challenging to distinguish between leptospirosis and dengue. Differentiating between leptospirosis and dengue is crucial since leptospirosis has a more favorable prognosis with early antibiotic therapy, whereas dengue does not have a specific treatment, although early detection is essential for close monitoring and cautious fluid management. Here, we highlight a case of dengue virus and leptospirosis coinfection in a female who presented with acute febrile illness, dyspnea, and altered sensorium, which progressed to multiorgan dysfunction syndrome, involving the neurological, respiratory, hepatic, and hematological systems.

## Introduction

Dengue fever (DF) and leptospirosis are global public health issues, especially in tropical and subtropical countries. Pathogenic Leptospira species [1] cause leptospirosis, a widespread anthropo-zoonotic infection, while any of the four dengue virus serotypes (DENV-1 to DENV-4) cause DF, an arthropod-borne viral disease [2].

Leptospirosis cases have increased in recent years as awareness and diagnostic facilities have improved, with the majority of cases occurring during the monsoon and post-monsoon seasons (July to November). During the same months, the highest incidence of dengue has been reported. Leptospirosis can cause life-threatening Weil's disease or a self-limiting acute febrile sickness. The illness was originally considered unimportant but is now a major public health issue. In India, DF is endemic and causes major morbidity and mortality. Self-limiting fever can lead to dengue shock syndrome or fatal dengue hemorrhagic fever. Leptospirosis and dengue may coexist in large numbers due to similar environmental conditions; however, there are few documented cases [3].

Early antibiotic treatment is effective for managing leptospirosis, while dengue is managed symptomatically [4]. The microscopic agglutination test is the gold standard for Leptospira identification; however, enzyme-linked immunosorbent assay (ELISA) is also commonly used to detect immunoglobulin M (IgM) antibodies [5]. Dengue IgM and immunoglobulin G (IgG) serology, along with non-structural 1 (NS1) antigen, are detected by the dengue combo test [6]. In addition to serology, real-time polymerase chain reaction confirms and detects the dengue virus. In patients presenting with less common forms of leptospirosis, the diagnosis is frequently either not considered or only discovered at autopsy [7]. This study discusses the radiographic results in patients with neurological symptoms caused by dengue and leptospirosis.

## Case presentation

A 51-year-old female patient presented to our medical center with complaints of altered sensorium, fever, and chills for the past five days, and dyspnea for the past two days.

A general examination revealed an average build, with the patient being moderately nourished. She was febrile with a body temperature of 100°F, and had a pulse rate of 128 bpm, blood pressure of 100/70 mmHg on inotrope, 94% SpO_2_, and respiration rate of 24 cycles/minute; dehydration was also noted. Positive findings included fever, tachycardia, hypotension, dehydration, tachypnea, and hypoxia.

During the systemic evaluation, on the central nervous system (CNS) examination, the patient exhibited signs of lethargy, irritability, noncompliance with verbal instructions, and movement of all four limbs. There was no neck stiffness. Kernig and Brudzinski signs were negative. Bilateral pupils reacted similarly to light. Bilateral extensor plantar reflex was noted. On respiratory examination, bilateral fine basal crepitations were present. On per abdominal examination, mild hepatomegaly was noted.

We investigated dengue, Leptospira, malaria, typhoid, and rickettsia, which are common causes of acute febrile illness with associated altered sensorium and mild-to-severe hepatic impairment. Day 1 reports (Tables 1, 2) suggested dengue and Leptospira coinfection fever with mild hepatitis and thrombocytopenia. Fluid resuscitation was started with crystalloids for dehydration and injection. Doxycycline 100 mg twice a day is given intravenously to treat leptospirosis.

**Table 1:** Blood investigations of the patient from D1 to D10 D, day; TLC, total leucocyte count; SGOT, serum glutamic-oxaloacetic transaminase; SGPT, serum glutamic pyruvic transaminase; RBS, random blood sugar

Parameters (normal limit)	D1	D2	D4	D6	D8	D10
Hemoglobin (12–16 gm/dL)	11.9	12.4	11.2	12.1	11.8	12.4
TLC (4,000–10,000/µL)	3,100	2,900	4,200	4,400	4,100	4,600
Platelets (150,000–410,000/µL)	22,000	16,000	48,000	82,000	146,000	186,000
Serum bilirubin (0.2–1.2 mg/dL)	1.66	1.88	1.64	1.32	1.30	1.28
SGOT (8–48 IU/L)	89	70	73	62	44	46
SGPT (7–55 IU/L)	77	74	70	64	52	54
Serum urea (17–49 mg/dL)	34	38	36	40	38	40
Serum creatinine (0.6–1.35 mg/dL)	0.68	0.89	0.74	0.86	0.82	0.90
RBS (up to 140 mg/dL)	106	128	104	136	138	138

**Table 2:** Additional investigations DENV, dengue virus; RT-PCR, reverse transcription-polymerase chain reaction; NS1, nonstructural protein 1

Test	Result
Dengue RT-PCR	Positive (DENV-2)
Dengue NS1 antigen	Negative
Anti-dengue IgG	Negative
Anti-dengue IgM	Positive
Leptospira RT-PCR	Positive
Leptospira IgM	Positive
Rapid malaria test	Negative
Widal test	Negative
Weil−Felix test	Negative
COVID RT-PCR	Negative

**Figure 1:**
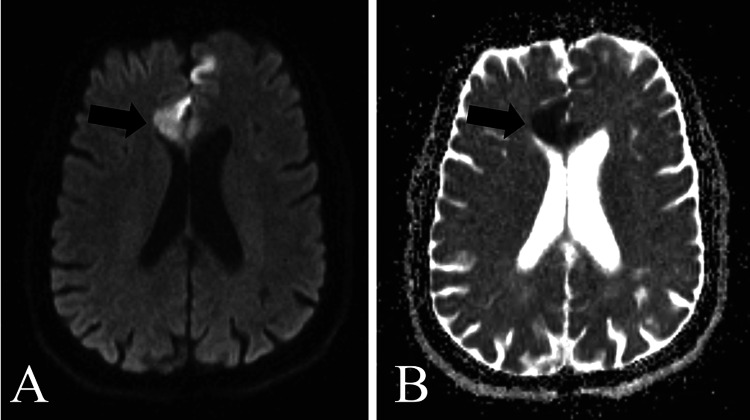
MRI of the brain (axial section) with diffusion-weighted imaging sequence showing areas of diffusion restriction in the bilateral frontal region (A) with corresponding low apparent coefficient value (B) MRI, magnetic resonance imaging

MRI of the brain (Figure 1) and CSF studies were conducted, both of which showed viral encephalitis changes.

**Figure 2:**
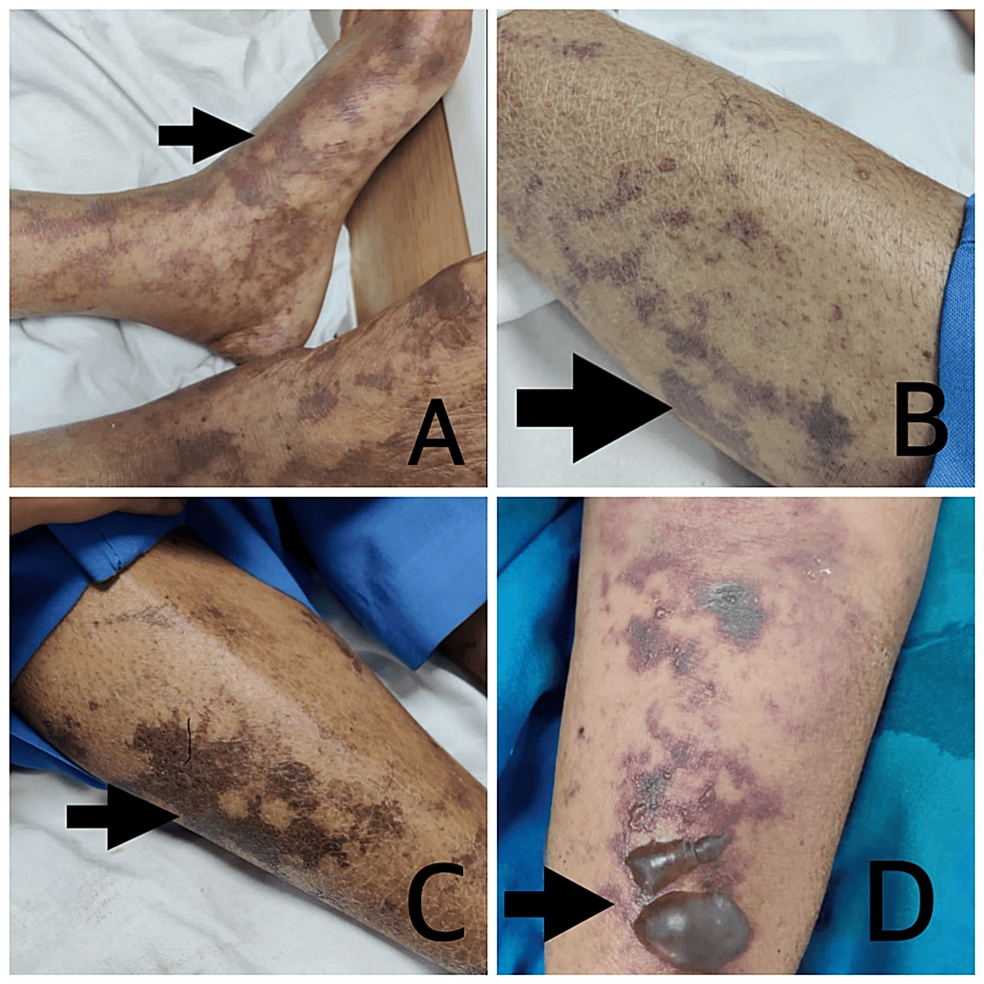
Images showing ecchymosis over the bilateral upper and lower limbs (A-C) and fluid-filled vesicles (bullae) associated with extensive subcutaneous edema (D)

In addition to two episodes of melena, there was worsening of the patient's thrombocytopenia, and the development of ecchymosis over both the upper and lower limbs occurred on day 3 (Figures 2A-2C). Furthermore, there was a great deal of subcutaneous edema on the front side of the left elbow and the area immediately around the forearm, as well as fluid-filled vesicles (bullae) all over the left upper limb (Figure 2D); the patient was transfused with single-donor platelets, fresh frozen plasma, and packed red blood cells.

An endotracheal intubation was performed on the patient immediately after she experienced respiratory distress on day 6, and mechanical ventilator support was started at the same time.

**Figure 3:**
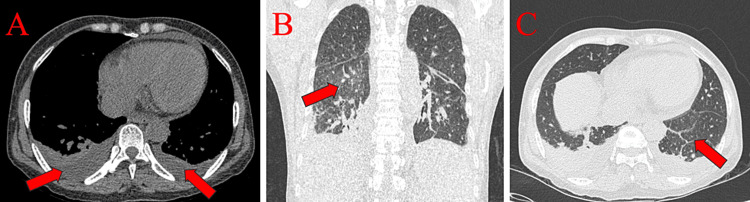
HRCT of the chest: (A) axial section showing bilateral pleural effusion, (B) coronal section showing ground-glass opacities in the superior segment of the right lower lobe, (C) axial section showing interlobular septal thickening in the left lower lobe

High-resolution computed tomography (HRCT) of the thorax was performed, which revealed bilateral pleural effusion and ground-glass opacities in the superior segment of the right lower lobe with interlobular septal thickening in the left lower lobe (Figures 3A-3C), suggestive of pulmonary edema. An undiluted infusion of furosemide 200 mg injection was started. The patient was kept on mechanical ventilation while arterial blood gases (Table 3) and other laboratory data were examined (Tables 1, 3).

**Table 3:** Arterial blood gas analysis of the patient ABG, arterial blood gas; pCO_2_, partial pressure of carbon dioxide; pO_2_, partial pressure of oxygen; SpO_2_, arterial oxygen saturation; sHCO_3_-, serum bicarbonate; FiO_2_, fraction of inspired oxygen; RA, room air

ABG	D6	D7	D8	D9	D10
pH (7.350–7.450)	7.30	7.36	7.40	7.46	7.43
pO_2_ (83.0–108 mmHg)	90	102	116	114	88
pCO_2_ (35.0–45.0 mmHg)	36	38	42	40	44
sHCO_3_- (18–24 mmol/L)	20	21	24	22	22
SpO_2_ saturation	91%	94%	98%	100%	98%
FiO_2_	0.50	0.50	0.50	0.30	RA (0.21)
PO_2_/FiO_2_	180	204	232	380	419

The patient was taken off the mechanical ventilator, and a T-piece experiment was performed before extubation. The patient was transferred to the ward for observation and then discharged. The follow-up went smoothly.

## Discussion

The global recognition of leptospirosis and dengue as tropical and subtropical acute febrile illness causes is growing. Healthcare providers in tropical regions often misdiagnose leptospirosis, a newly identified zoonosis, as dengue, malaria, scrub typhus, or typhoid [8,9]. Jaundice, thrombocytopenia, renal failure, and bleeding can result from leptospirosis and dengue, which can be moderate or fatal. Major causes of acute febrile illness, dengue (10%) and leptospirosis (37%), are becoming well known [10,11].

Individually, dengue and leptospirosis constitute a management difficulty. Coinfection has grown to be a major issue. Both leptospirosis and dengue happen during the wet season, and laboratory confirmation of the pathogen is frequently slow [12]. Numerous studies have misidentified leptospirosis as dengue in endemic locations [13,14]. Public health professionals and clinicians can start the proper antimicrobial medicine and reduce mortality by distinguishing dengue from leptospirosis and detecting coinfection in endemic communities [15].

Prior research by Brown et al. found dengue and leptospirosis coinfections; 2.5% of 314 dengue IgM-positive samples also had Leptospira IgM [16]. In patients with dengue-like symptoms and no serological evidence of active primary dengue, leptospirosis should be considered. Thus, rapid leptospirosis testing may help. These steps would detect leptospirosis early and reduce fatalities.

The clinical overlap between DF and leptospirosis may lead to misdiagnosis or underdiagnosis of mixed infections. Both can cause acute febrile illness with chills, myalgia, headache, backache, abdominal pain, and anorexia. A cutaneous rash suggests DF, but conjunctival suffusion suggests leptospirosis. Leukopenia, lymphadenopathy, and hepatomegaly are more common in DF and leptospirosis, whereas thrombocytopenia may occur early in both [17]. 

Recent serum IgM ELISA-confirmed dengue infection, fever, aches and pains, thrombocytopenia, and dengue virus endemicity in India are all warning signs of DF. To establish dengue viral encephalitis, neurological symptoms, such as altered mental status, headaches, seizures, nystagmus, focal neurological abnormalities, Babinski's sign, and vertigo, are combined with dengue symptoms. CNS involvement in DF can be accurately and non-invasively assessed using MRI [18].

In a study conducted by Rodrigues et al. on lung involvement in DF and CT imaging [19], it was noted that due to respiratory symptoms, 29 (1.43%) of 2,020 dengue cases underwent chest CT scans. Out of 58.6% of patients with abnormal chest CT scans, pleural effusion was the most prevalent finding in 55% and the only finding in 35%. The most common lung parenchymal involvement finding was ground-glass opacity in 27.5% of individuals. Lung consolidation occurred in 20.6%. Interlobar septal thickening and non-specific airspace nodules were found in two (6.82%) patients.

Clinical outcomes are best with early treatment. Multiorgan failure can occur in severe situations. Renal, hepatic, hematologic, and CNS problems require supportive therapy and cautious monitoring. Due to early detection and medical treatment, DF seldom kills more than 1% of patients. Treatment reduces severe dengue mortality to 2-5%, but if left untreated, it might reach 20%. Symptomatic and supportive dengue treatment frequently works [20].

## Conclusions

It is important to consider the likelihood of dengue and leptospirosis coinfection as high prevalence is noted, especially in endemic areas where the conditions favor the spread of both organisms. It is vital to differentiate the non-specific imaging results of dengue from those of other disorders that have similar appearances, which may coexist and occur in the same epidemiological context. The primary focus of management is on attentive, supportive care and thorough monitoring of the patient's condition. Early detection and management are critical to averting negative consequences in these circumstances.
